# Improved Arabic query expansion using word embedding

**DOI:** 10.1038/s41598-025-28758-0

**Published:** 2026-01-22

**Authors:** Yaser A. Al-Lahham, Sattam Almatarneh, Kaznah Alshammari, Mutasem Al-Smadi

**Affiliations:** 1 Computer Science Department, Faculty of Information Technology, Zarqa University, Zarqa, 13110 Jordan; 2 Department of Data Science and AI, Faculty of Information Technology, Zarqa University, Zarqa, 13110 Jordan; 3 Department of Information Technology, Faculty of Computing and Information Technology, Northern Border University, Rafha, 91911 Saudi Arabia; 4 Department of Management Information System, College of Applied Studies and Community Service, Imam Abdularahman Bin Faisal University, Dammam, 34212 Saudi Arabia

**Keywords:** Arabic information retrieval, Query expansion, Word embedding, Pseudo-relevance feedback, Word2Vec, GloVe, SkipGram., Engineering, Mathematics and computing

## Abstract

Word embedding enhances pseudo-relevance feedback query expansion (PRFQE), but training word embedding models takes a long time and is applied to large datasets. Moreover, the Arabic language, which has rich morphology, dialectal variations, and a lack of high-quality linguistic resources, training embedding models need special processing. This paper proposes using a representative subset of a dataset to train such models and defines the conditions of representativeness. Using a suitable subset of words to train a word embedding model is effective since it dramatically decreases the training time while preserving the retrieval efficiency. This paper shows that a subset of words is derived from an Arabic dataset, which consumes 10% of the training time of the whole dataset, and preserves retrieval efficiency. The trained models are used to embed words for different scenarios of Arabic query expansion, and the proposed training method shows effectiveness as it outperforms the ordinary PRFQE by at least 7% Mean Average Precision (MAP) and 14.5% precision improvement at the 10th returned document (P10). Moreover, the improvement over not using the query expansion is 21.7% for MAP and 21.32% for the P10. The results show no significant differences between using different word embedding models for Arabic query expansion.

## Introduction

The main problem with query answering is that most users submit short (less descriptive) queries or use words that differ from those used in documents^1^. Furthermore, writing informative user queries is still tricky, and it is hard to represent their needs^2^, in addition to the problems encountered when dealing with natural languages, such as synonymy and polysemy. The Arabic language has an additional problem since it is a fluctuating language; some words could have the same shape but have different diacritics, so they represent different meanings, which leads to word ambiguity^3^. Query expansion using relevance feedback, either user feedback or pseudo-relevance feedback, was proposed to expand users’ queries by adding (or embedding) other words most likely to have the same meaning as query words and resubmit the query^4^. These embedded words should be selected to increase the probability of occurring in more relevant documents to that query, improving retrieval^5^. In most cases, query expansion systems add synonyms, hyponyms, and hypernyms of the words of the original query to improve precision and recall^6^. Word embedding uses a large dataset to determine the context of some words, and each word is represented as a vector of values, such that similar words have closer vectors^7^. These vectors are formed by positioning words so that their position corresponds to their semantic properties^8^. Most methods that use word embedding to expand users’ queries rely on the assumption that words co-occur in the same context and mostly have the same semantics^8^ or the ”distributional hypothesis.” Word embedding techniques could be prediction-based, depending on the word context, such as (Continuous Bag of Words CBOW, SkipGram)^9^, and FatText^10^, trained on character n-grams, or count-based that depends on global word-word co-occurrence beside the local context of a word to determine words’ vectors^11^, an example of count-based is GloVe^12^. Moreover, queries could be expanded using local or global word embedding. Local embedding query expansion relies on adding selected words from the pseudo-relevant documents of the query, while global embedding incorporates semantically related words from all over the corpus^13^.

As words in the same context co-occur with query terms and are embedded to enhance retrieval, other problems could be raised due to this word embedding. Query drift is a possible problem, which can occur because of adding new words to the query. It could be possible that those words co-occur with too many other words, and some are common words that are most likely to have less significance to be added to the query. Adding these words to the query will return a larger number of other documents that are not relevant to the query. This situation will increase false negative documents and reduce precision, or result in minor precision improvement. Recent works attempted to minimize the effect of the query drift on Arabic query expansion; for example, in^14^, the authors applied swarm optimization algorithms to get the most similar set of terms to a query term. On the other hand, in^15^, Deep Median Networks (DMN) were incorporated with the CBOW embedding to improve the Arabic Query Expansion. However, the effectiveness of word embedding is sensitive to the choices made during the training phase, where the choice of training corpus and term normalization affects the retrieval efficiency, as it is found that the result when normalizing terms before training is different from normalizing after training^5^. Therefore, word embedding models could produce biased word vectors as they are sensitive to the training corpus. Moreover, these models do not distinguish between similarity and relatedness^16^. Another issue with these word embedding models is that some words of opposite meanings could have the same context and, consequently, be reported as similar terms in each vector^17^. Recently, many works on word embedding have been proposed to enhance Arabic information retrieval using pseudo-relevance Arabic query expansion. Some works depend on determining the distribution of words in the pseudo-relevant document; for example, Abdelkader El Mahdaouy et al. in^3^. Other researchers included the morphological annotation of Arabic words in the embedding procedure, such as Rana Salama et al.^18^. They incorporated the POS tags of words before word embedding and the lemma of each word to leverage the morphological and semantic similarity of words to the query words. Hiba AlMarwi et al.^1^ used WordNet to include semantically similar words to Arabic words before embedding, and they used Particle Swarm Optimization to calculate the weight of the term. They expanded the query by the top-m weighted words. Arabic Word Net is also used by^13^ to identify the concepts of text to be used to expand queries, in addition to simple and compound words.

This paper proposes an Arabic query expansion word selection to improve Arabic query expansion by focusing on a subset of representative words to train the word embedding instead of using all words of the dataset. These words are more likely representative of the dataset semantics than selecting all the words while reducing the training time and the space needed to store words’ vectors. This proposal attempts to solve query expansion’s inherent problems, such as document cleansing, indexing, and ranking^6^, which is essential to be more accurate since the pseudo-relevant set of documents used to expand a query is selected using a ranking algorithm. Moreover, some methods rank the selected words according to statistical or semantic properties^19^. The trained word embedding model is used for query expansion by applying different scenarios, such as embedding words of the original query or embedding the expanded query, expanding using the top-weighted words of each document, or using top-weighted words of all top-ranked pseudo-relevant documents.

Finally, word embedding models work at the word level, so techniques must be used to make these models effective when dealing with languages with rich morphological structures^20^, such as the Arabic language, which motivated this work. The rest of the paper is organized as follows: Sect. “Related work” gives a review of the related work and concludes the gaps that motivate this work, a brief description of word embedding is given in Sect. “Word embedding”, the proposed method of Arabic query expansion is illustrated in Sect. "Proposed Arabic query expansion" followed by the implementation and evaluation of this method in Sect. "Implementation and evaluation"; finally, the overall work is concluded in Sect. “Conclusion”.

## Related work

Query expansion aims to add more terms to the user query to increase the probability of having a match between possible relevant documents. Several research efforts tried to achieve this goal by increasing this probability using statistical methods (or contextual word embedding). However, they mainly rely on language models, in which a word can be predicted given its context. Other researchers used semantic methods to add these terms. They used pre-defined structures of semantic relationships. In this case, words’ semantics could be determined using a thesaurus or an ontology^21^; and^22^ are examples. On the other hand, some researchers combined these two approaches and proposed hybrid query expansion methods, as in^1^.

Statistical-based methods use terms from the returned documents or the pseudo-relevant set to expand the users’ queries. In contrast, the semantic methods use semantically related terms to query terms and documents. The relatedness is determined using some knowledge graph. In this case, the terms used to expand queries may not be included in the documents of the corpus, so to get better results, they need to expand the documents’ terms in the same way, or what is called dense retrieval^23^. The following works are examples of statistical methods.

El Mahdaouy in^3^ and^19^ proposed a query expansion method using word embedding similarity to improve Arabic information retrieval. They extracted terms from the top-ranked documents of the pseudo-relevant set according to their distribution in the documents, besides their similarity to the original query terms. They used the TREC 2001/2002 collection to train the Word2Vec and other data sets, including Wikipedia and tested their proposal on the TREC 2001/2002 collection.

Farhan YH et al.^24^ proposed expanding the query instead of expanding each term. They incorporated several neural network layers to average the word vectors of a query to get its semantics. The authors extended their proposal in^25^ by using deep median networks to overcome the problem of semantic drifting within an expanded query by incorporating the median vectors into the candidate expansion terms. Ibrahim Muwad et al.^26^ proposed a query expansion based on a bi-gram model, which expands the query by adding similar bigrams to the query terms. There is no pseudo-relevance feedback. The following examples of recent works present ontology-based semantic research efforts for query expansion.

Ashraf Mahjoob et al. in^21^ used WordNet to expand query terms using two methods; the first one expands all terms of queries, forming a single expanded query. The second method used the expanded terms to form a set of queries and combined the results of these queries to get the final relevant document list. Shivani Jain et al.^22^ proposed a fuzzy ontology-based query expansion, combining global and domain-specific ontologies. The resulting fuzzy ontology determines the semantically related terms to the query terms. The weight of terms is determined using a fuzzy function depending on several types of semantic relationships.

Hybrid methods expand queries by lexical words and their related words in an ontology or thesauri, for example, Hiba ALMarwi in^1^ proposed a hybrid approach for query expansion, which depends on Particle Swarm Optimization to infer semantic properties (and avoids query drift) out of possible statistical query expansions, which are obtained using word embedding, WordNet, and term frequency.

Ayyoob Imani et al.^27^ improved the retrieval by applying a classifier on the word embedding results to select words in the context of a given word correctly. They applied the proposed method to TREC English collections. Rana Aref Salama et al.^18^ used embedding similar words, embedding morphological annotations, and representing word vectors as lemma forms to enhance query expansion. They evaluated the proposed method on different NLP applications. Ayat Elnahhas in^28^ used an Arabic dictionary and words from the pseudo-relevance feedback to expand queries, and Hiteshwar Kumar in^29^ used web knowledge for query expansion. Said El Alaoui and Khalid Zidani^30^ proposed a hybrid method for query expansion by using Part-of-Speech and Arabic WordNet to extend the pseudo-relevance expanded query. They aimed to answer questions by converting them into queries and expanding these queries to improve their results. Another approach uses contextualized query expansion using a chunk of text instead of the whole document, for example, Zhi Zheng et al. in^31^.

Currently, some methods have enhanced word embedding through other means, such as optimization algorithms, neural networks, and large language models. Farhan et al. in^15^, proposed a method that integrates the Deep Median Networks DMNs with different existing IR models: BM25, EQE1, and V2Q. The methodology involves using a pre-trained Word2Vec model on the TREC 2001/2002 Arabic newswire corpus to represent query terms as vectors, calculating the median vector with DMNs, and then using cosine similarity to find and add the most similar terms for query expansion. The best result was achieved by combining Embedding-based Query Expansion (EQE1) with the DMNs. Allahim and Cherif in^14^, the authors suggest a framework to enhance the semantics of an expanded query that utilizes language ontologies and incorporates meta-heuristics optimization algorithms, which optimize term selection of the expanded queries. While the results achieved were promising, challenges remain in ensuring the seamless integration of diverse linguistic resources and maintaining efficiency in processing expanded queries. Mohammed Alqarni in^32^ applied the Large Language Model (LLM) embedding for the semantic search of the Quranic text. While the used embedding enhanced the results of some selected queries, the author found and discussed some limitations of applying the LLM embedding to the semantic search. Although the recent works described above enhance the query expansion, they may face challenges in implementation due to their computational complexity and high resource demands.

In conclusion, PRFQE is an effective method to improve retrieval, but it could be enhanced using different approaches, as presented above. Mainly, using a neural network model for word embedding is effective but needs a large training data set, which consumes more time^11^. On the other hand, an extensive empirical study should be conducted to determine the optimal settings, such as the number of pseudo-relevant documents, top selected terms, global (corpus-based scale) or local (pseudo-relevant documents scale) term similarity, and what to expand. Expand queries only or expand documents and queries; these factors were found to have considerable influence on the retrieval effectiveness, as reported in^5^. The effectiveness of word embedding motivated the research of this paper, which proposes a simple method of word embedding PRFQE by limiting the training of the embedding model to a specially selected representative subset of words and examining different schemes of query expansion. This method shortens the time needed for training and, at the same time, keeps the retrieval efficient.

## Word embedding

Word embedding can be classified as prediction-based, which depends on analyzing the local context of a text, and count-based models that analyze the overall dataset for counts and frequencies of words^11^. Some researchers refer to these models as text-based models since other later models were proposed to enhance these models by incorporating an ontology, as indicated in^20^. This section will focus on text-based models since it is used in this research.

Word2Vec proposed two models, namely the Continuous bag-of-words and SkipGram, as prediction-based models. The former predicts the central word by examining some context, i.e., it maximizes the probability of a word to be in some context^9^. On the other hand, SkipGram is used reversely as a central word to predict its context^11^. Word2Vec produces a vector for each word that combines all the senses of that word, so it is a non-contextualized model^33^. For better prediction, FastText^10^ proposed an improvement of the SkipGram instead of word embedding to n-gram character embedding. N-gram character embedding could be better used for fluctuating languages, where a word could contain information in its parts. Moreover, infrequent words that are generally analyzed could be better estimated, which leads to improved lexical tasks^7^.

Co-Occurrence Matrix, or count-based models, this model considers the global representation of a corpus to obtain the co-occurrence matrix of words. The entries of this matrix could be obtained using a Singular Value Decomposition SVD, which tackles problems of sparsity of high-dimensional embedding matrices and disparity of words’ distribution, simplifying complex computations^7^. C0-Occurrence could better be found by comparing the co-occurrence of a word x with a word y, more than it co-occurs with a stop word, for example^34^.

GloVe is an embedding model that considers the ratio of word co-occurrence instead of the actual number of co-occurrences of two words globally in a corpus. It is found that the ratio of the probability of a word co-occurring with other words to the probability of another word co-occurring with the exact words represents a meaning scale. This model shows superiority over other models in some NLP applications^11^.

A transformer-based model and contextualized models, such as BERT^35^, use attention to give high weights to important words in the context of some words and ignore others. In this model, each sentence begins with a ”classification” modifier, ”CLS,” and ends with a separator ”SEP.” The model reads sequences (sentences) several times in both directions, and the output is a vector for each token. A token can be predicted by substituting a term in a sentence with a MASK token, and the model returns multiple alternatives of that position of the MASK in a given sentence. Recently, ChatGPT (https://openAI.com/chatgpt), developed by OpenAI, used transformers for advanced human-like interactive conversation between the system and the user. Recently, pre-trained large language models (LLMs) have been used to represent both documents and queries as vectors of a latent dense layer, which is called dense retrieval^36^.

## Proposed Arabic query expansion

This section explains the proposed Arabic query expansion based on word embedding. The method includes training the word embedding model using a subset of words that represent the overall dataset, with a detailed description of the approach of selecting this representative subset and the rationale behind this selection. The other topic that is explained in this section is how to use the trained word embedding to enhance the pseudo-relevance feedback query expansion (PRFQE).

### Training the word embedding model

In this proposal, the word embedding model is trained using a variant of each sentence in the dataset. Each variant of a sentence is composed out of specially selected words. The set of selected words should be small enough to make the system efficient and representative enough to share the same contexts with other words with high probability, since the training dataset considerably influences the effectiveness of the word embedding model^5^. To effectively measure the extent to which a selected subset of words represents the total set, we must test its distribution across all documents during index construction; the minimum requirement for this subset (defined by Definition-1) is that all documents be included in the index (i.e., no document is unindexed), as a greater number of unindexed documents directly weakens the subset’s representational quality. Furthermore, the selected representative subset must also preserve the context of the sentences within these documents, a condition that can be validated by calculating the ratio of words from the overall dataset that appear within the context of any member word in the selected subset.

#### Definition 1

Given that the vocabulary of some dataset language is *V*, and a selected subset of terms $V_s$. If *V* is used to train the word embedding model, the subset $V_s$ is considered *representative* if it satisfies the following two conditions:

- For each document *D* in the corpus, at least one word $w_i$ exists in $V_s$ such that $P(w_i|D)> 0$.
- For each word $w_i$ in $V_s$, there exists a word $w_j$ in *V* where the probability of $w_j$ to co-occur with $w_i$ in a context $X_{w_i}$: $P(w_j|w_i) = 1$.

The first condition of definition 1 could be used to preserve an essential property of the embedding model, which is the ”good geometry” property; a model satisfies good geometry as it distributes the words of documents around the selected subset of words of that dataset. The second condition of definition-1 states that a word of the subset in a document makes the model ”reliable” since it guarantees that each word of the representative subset co-exists with at least one word of the corpus in at least a single context Xwi. Good geometry, reliability, and other properties of the word embedding model are explained in^7^. Since the result of a word embedding depends on the dataset^5^, the selected subset of words, which produces shorter sentences, is expected to bring more words of similar semantics to co-occur in a sentence. Moreover, the dimension of each word is expected to be smaller, which could enhance the semantic relationship between words in the same context that depends on the dimension size of the word embedding and the word frequency^37^. The selection of a representative subset could follow the same approach used for feature selection, which is based on some linguistic intuition and an empirical procedure, as presented by^38^. Linguistic intuition means that this subset demonstrates the semantics of the overall dataset. The proposed selection criteria of a representative subset of an Arabic dataset follow the linguistic intuition given in^39^. That linguistic intuition indicates that the subset of AL-Definite words could be selected as a representative of a dataset. To determine that this subset of Arabic words can be considered representative of an Arabic dataset, it should satisfy the conditions of definition 1. As illustrated by^39^, the AL-Definite words were distributed over all of the datasets, since –for example- these words indexed all of the documents of the TREC 2001/2002 corpus; i.e., each document contains a subset of AL-Definite words, which satisfies the first condition of Definition-1.

Moreover, it has been found that 98.3% of the words (after stemming) of the TREC 2001/2002 co-occur within a three-word-sized context around an AL-Definite word. Intuitively, if the context of a word is widened, then more words could be included within a word’s context in this subset, which satisfies the second condition of definition 1. Additionally, the researchers in^39^ found that using these definite words and words before them, for example, improved the retrieval over using all words of the corpus, making this selection satisfy information retrieval applications, which satisfies the assumption in^38^ that feature selection is an application-dependent task.

**Fig. 1:**
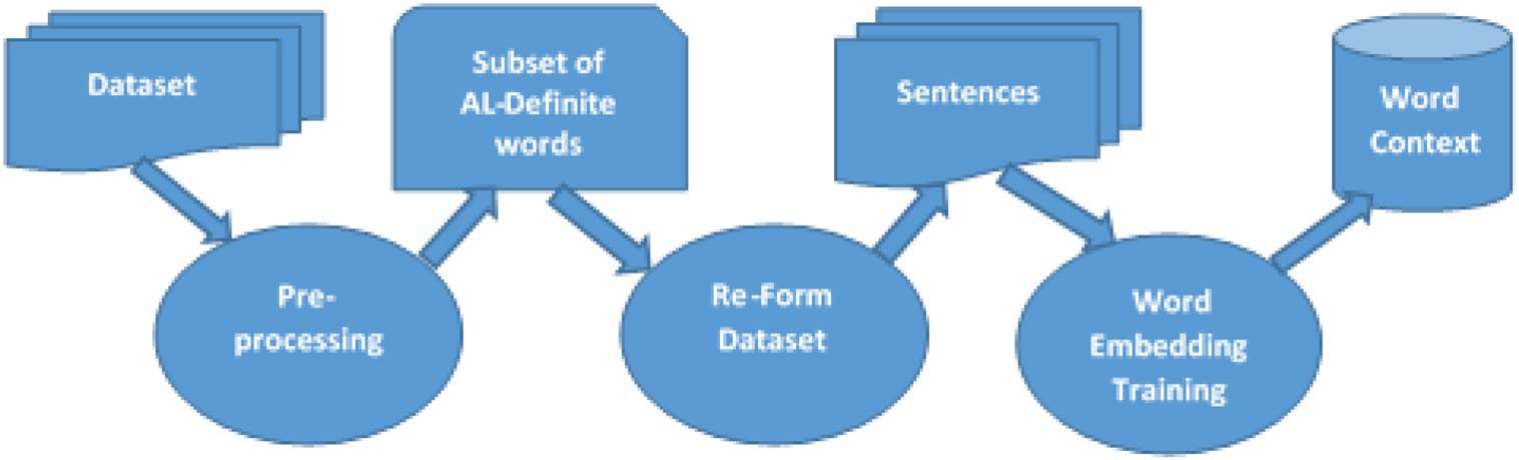
Word Embedding Model.

The word embedding model is trained using the selected subset of representative words, as follows: divide the dataset into sentences, each sentence is composed of only the definite words, either stemmed or not, then the top similar words to each word are stored in a table, the implementation details are presented in the implementation and evaluation section, and the whole process is illustrated by Fig. 1.

To theoretically estimate the relationship between training the embedding model using the whole dataset and with a representative subset of that dataset, we will use the approach explained in^5^. In that approach, the Jaccard average similarity between two embedding models, each model resulting from training the same word embedding by a different dataset, and the correlation between these two resulting models is calculated. The subset of the selected words is considered representative if the computed similarity between the model trained by this subset and the model trained by the whole dataset is high and the normalized correlation between them is close to one.

The set of words and their vectors resulting from training a word embedding on a dataset is called the embedding space. Considering the embedding space $E_V$ of the model trained by the whole dataset $V$, and (by applying the second condition of Definition 1, $E_{V_s} \subseteq E_V$) the embedding space of the model trained by a representative subset $V_s$, the intersection between them is $E_{V_s}$.

For each word $w$ in $E_{V_s}$, the intersection of the context of $w$ in both embedding spaces is its context in $E_{V_s}$, since the context of $w$ in $E_{V_s}$ is a subset of its context in $E_V$. Therefore, the union of the two contexts of $w$ is its context in $E_V$.

The Jaccard similarity ($J_i$) between the embedding spaces trained by both $V$ and $V_s$ is given by Equation 1:

The set of words and its vectors resulting from training a word embedding on a dataset is called *embedding space*. Considering the embedding space $E_V$ of the model trained by the whole dataset $V$, and (by applying to the second condition of Definition 1, $E_{V_s} \subseteq E_V$) the embedding space of the model trained by a representative subset $V_s$, the intersection between them is $E_{V_s}$. Therefore, for each word $w$ in $E_{V_s}$, the intersection of the context of $w$ in both embedding spaces is its context in $E_{V_s}$, since the context of $w$ in $E_{V_s}$ is a subset of its context in $E_V$. Consequently, the union of the two contexts of $w$ is its context in $E_V$. The *Jaccard* similarity $J_i$ between the embedding spaces trained by both $V$ and $V_s$ is given by Equation (1):

1 $$\begin{aligned} J_{i}\left( E_{V},E_{V_s} \right) = \frac{1}{|E_{V_s}|}\sum _{w \in E_{V_s}}^{}\frac{|X_{i}^{E_{V_s}}|}{|X_{i}^{E_{V}}|} \end{aligned}$$

where $X_{i}^{E_{V}}$ is the $i^{th}$ context of the closest words of the word $w$ in the embedding space $E_V$, and $X_{i}^{E_{V_s}}$ is the corresponding $i^{th}$ context of the closest words of $w$ in the embedding space $E_{V_s}$, where $|X_{i}^{E_{V_s}}| \ge 0$ and $|X_{i}^{E_{V}}|> 0$.

It is clear that the conditions of Definition 1 lead to higher values of $J_i$, since they ensure that the words of the selected subset are distributed over all of the contexts of the whole dataset.

The correlation between the embedding spaces produced by the model when trained by the whole dataset and by a representative subset should be high, as there is a complete overlap between the two spaces because $V_s$ is included in $V$. The correlation between $E_{V_s}$ and $E_V$ is given by Equation 2:

2 $$\begin{aligned} \begin{aligned} C_{i}\left( E_{V},E_{V_s} \right) =&\frac{1}{|E_{V_s}|}\sum _{w \in E_{V_s}} \\&\frac{1}{2}\left( 1 + C\left( X_{i}^{E_{V}}(w),X_{i}^{E_{V_s}}(w) \right) \right) \end{aligned} \end{aligned}$$

It is easy to show that the value of correlation is high because it is computed between the contexts of the same word in both context spaces. From Equation 2, it can be noticed that the summation indicates that a word $w \in E_{V_s}$ is also included in $E_V$.

The explanation given above indicates that a representative subset of a dataset, which satisfies Definition 1, could be effectively used to train the embedding model without losing the accuracy of the query-expanded retrieval system and at a shorter training time.

The proposed method of selecting a representative subset of a dataset is used to train the word embedding model. As word embeddings predict the context of a word, the words within a successfully selected representative subset should have predicted contexts similar to the contexts in the whole dataset from which they were picked. Consequently, the effectiveness of the subset selection could be validated by comparing the predicted context returned by a word embedding model of a word with the actual context of that word in the dataset.

After training the word embedding model, a query could be expanded using different scenarios for query expansion and embedding. Scenarios differ regarding the time of query word embedding: before or after pseudo-relevance expansion. Furthermore, which word embedding is applied: on the top-selected words of each pseudo-relevant document or the top-selected words of the combination of all pseudo-relevant documents? The following subsections explain each of the three embedding scenarios.

### Pseudo-relevance feedback

The proposed query expansion scheme depends on determining the top-most similar words to each query word using word embedding. The query is pseudo-relevant expanded after getting the returned documents of the first run; as a result of that query, selecting $d$-top-ranked documents, and out of each selected document, select $m$-top weighted words. For each selected word $w$, get the $t$-top similar embedded words; the query is then expanded using different alternatives, as explained later in this section, and the query is resubmitted to the system for a second run. The maximum number of words that are embedded in each query could be estimated by Equation (3):

3 $$\begin{aligned} M_t = d \cdot m + t \cdot |Q| \end{aligned}$$

where $M_t$ is the maximum number of expanded words to the query $Q$, and $|Q|$ is the number of words in $Q$. This equation assumes that word embedding is applied to the original query, not to the expanded query.

These three parameters ($d$, $t$, $m$) are tuned to give more chance to returning more relevant documents to a query.

**Fig. 2:**
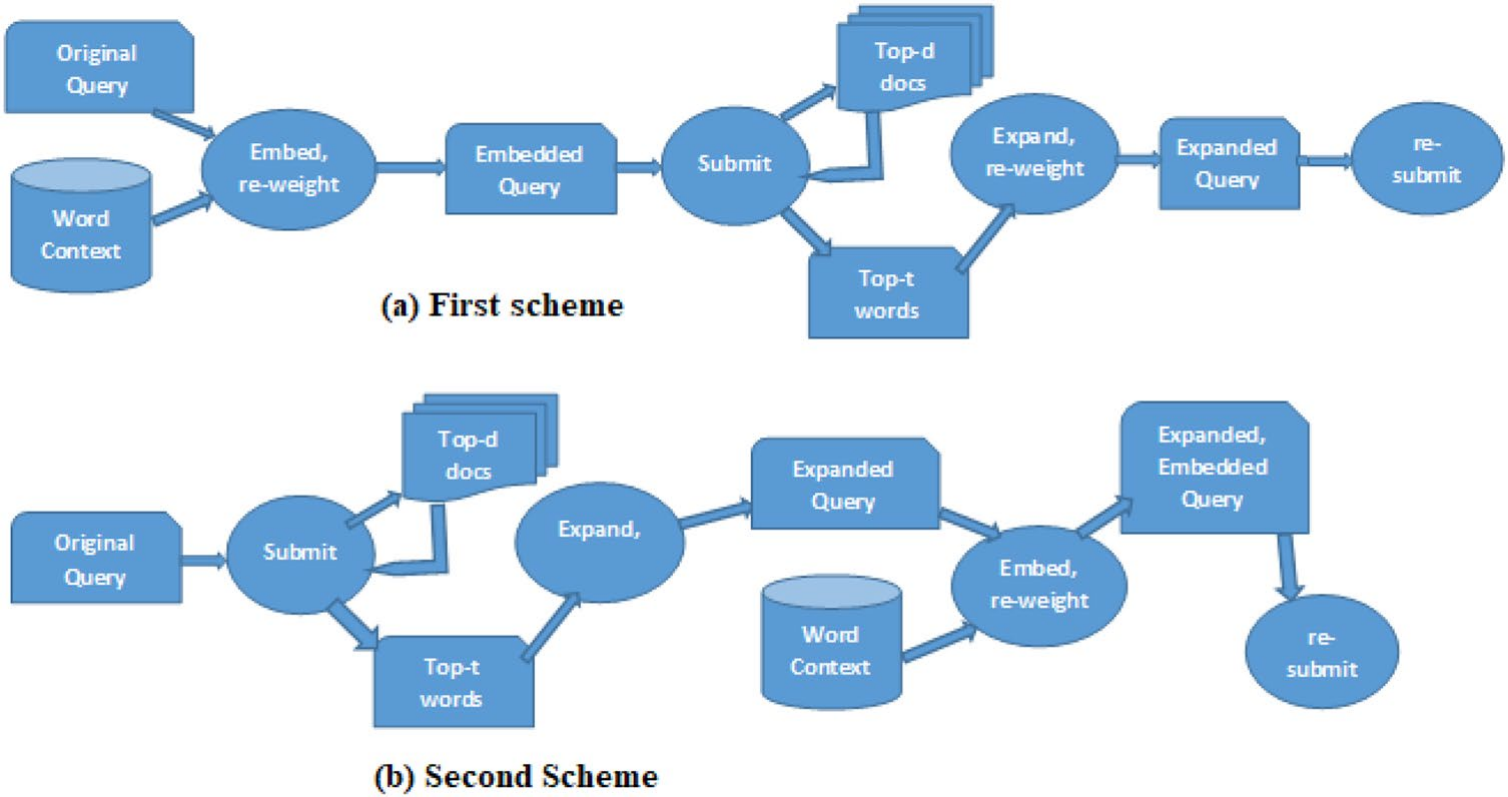
Word Embedded PRFQE Schemes.

Tuning these parameters should take care of the following issues:

1. The number of the top-selected pseudo-relevant documents (*d*) should be large enough to highly represent query semantics and small enough to prevent other false positive documents from being included in the pseudo-relevant set of documents since it is found that selecting a small number of pseudo-relevant documents could get bad results in case of ”Synonymy or Polysemy types of queries”^29^.
2. As the number of top-weighted words (*m*) selected from each pseudo-relevant document gets larger, more unwanted words could be added to the query since lower weights of words (in a document) relatively indicate a weak representation of that document, and adding other words in the context of such lower weighted words (using word embedding) is expected to change the semantic of the query.
3. Finally, as *t* gets larger as the similarity to the extended words gets lower since words that share the same context are not necessary to have the same semantics in an embedding model^8^, and adding embedded words from higher dimensional vectors gives more chance to change the semantic of a context because increasing the dimension of the word embedding has adverse effects on the model^37^.

In this paper, we will use the following two schemes of query expansion and embedding:

- The first scheme is to embed words of the original query, get query result, get the top-*d* similar documents, pseudo-relevant expand the embedded query, re-weight words, and resubmit the query; this scheme is represented by Fig. 2 (a).
- The second scheme is to submit the original query, get query results, get top-*d* similar documents, pseudo-relevant expand the query, embed the expanded query, and resubmit the query; the second scheme is described in Fig. 2 (b).

On the other hand, two methods of selecting expansion words from the top-*d* documents are used:

1. For each document of the top-*d* returned documents, choose the top-*m* weighted words
2. An alternative method of selecting these expansion words could be achieved by selecting the top-*d* relevant documents, mixing all of the selected words of all documents in one list, re-weight repeated terms, descend sorting the list, and select top-weighted words from the list as computed in Equation 3.

Having the embedding model trained using the representative subset of words, we now discuss how this selection will improve the retrieval of query expansion methods and apply the assumptions and schemes of pseudo-relevance feedback query expansion. Initially, word embedding query expansion depends on the assumption that the original query words have similar words in other contexts, which could be included in more relevant documents to that query^24^.

According to this proposition, and after applying word embedding, the following possible cases could increase the chance of returning more relevant documents to a query:

A. Adding top-*t* words of pseudo-relevant documents to the query will increase the similarity between these documents and the query, keeping these documents in the top-*d* similar documents, which preserves the relevant returned documents.
B. The added top-*t* words (from each top-*d* returned document) that weren’t included in the original query could be included in other relevant documents not returned in the first run (before query expansion). These documents could be included in the top similar returned documents, increasing the precision of that query.
C. Adding embedded words to the original query could have the following effects on the returned documents:
  1. The added embedded words could be included in relevant documents that did not return in the first run, giving these documents a chance to be in the top-similar documents, which will –also– increase the precision of that query.
  2. The added embedded words could be included in the relevant returned documents, increasing the similarity of these documents to that query. Thus, these documents will rank higher in the returned top-similar documents, positively affecting the precision at lower recall levels.
  3. The added embedded words could increase the frequency of some query words, as they could be repeated in the original query, giving higher weights to these words. It is reasonable to give these words higher weight since repeating such words in the context of the query’s words provides more evidence that the embedded words share the same semantics as the query.
  4. The order in which the embedding is applied affects the result of query expansion. If we only embed the original query (the first scheme), the context of query words will be exclusively added, so returned documents are expected to have the same semantics as query words. If the second scheme is used to apply embedding to the pseudo-relevant expanded query, the returned documents will have contexts of similar semantics to both the query words and the semantics of the selected top-*t* words of other pseudo-relevant documents. The second scheme is expected to add more true-positive documents while simultaneously adding false-positive similar documents to the result of the second run. This occurs because many embedded words of different semantics (than the original query words) could be added. This order of embedding could negatively affect retrieval when the top-*t* list becomes longer, as the chance of adding unwanted words increases with more words added to the query.

In the next section, the details of implementing the proposed pseudo-relevance expansion schemes are presented, where different scenarios are tested to show the effectiveness of the proposed training of the word embedding model using a subset of words.

## Implementation and evaluation

This section presents the implementation and testing details of the proposed query expansion based on word embedding. The first part of the implementation deals with the word embedding training dataset, such that two of the word embedding models, namely the SkipGram^9^ and the GloVe^12^ models, are trained using two sentence forms. The first form includes all the words in the dataset, and the second form includes only the AL-Definite words (Arabic words with the prefix ’AL’). This part of the implementation aims to test the effect of using a representative subset of the dataset against the whole dataset, which was explained in Sect. "Training the word embedding model".

**Table 1:** Training text forms description.

**Text form**	**Description**
**ALLNOSTEM**	Training the embedding model using all of the text (whole dataset) without stemming.
**ALNOSTEM**	Training the embedding model using only AL-Definite words without stemming.
**ALLSTEM**	Training the embedding model using all of the text with Light10 [38] stemming
**ALSTEM**	Training the embedding model using AL-Definite words with Light10 stemming.

The second part of the implementation and testing concerns the application of PRFQE schemes, as explained in Sect. "Pseudo-relevance feedback". The word embedding models were trained using the TREC 2001/2002. TREC 2001/2002 is an Arabic dataset of 383872 Agence France Presse (AFP) newswires collected between the years 1994-2000. It is a large-scale, publicly available test collection for the Arabic language. It provided a standardized benchmark for evaluating and comparing different Arabic IR approaches, including 75 user needs (queries) with judgments. Each user needs has three sections: title, description, and narrative. News documents are preferred for evaluating IR models because they have a standardized format, consistent quality, and broad, factual content. Moreover, newswires are still used for recent applications, as^40^. Four options of the training text were used, where two options (whole dataset, representative subset) are combined with the other two options (stemming, no stemming options), as presented in Table1.

**Fig. 3:**
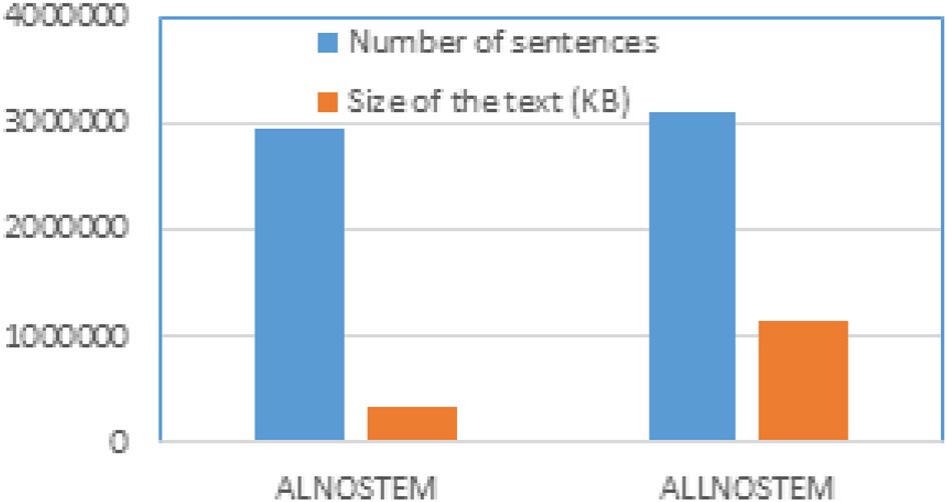
Number of sentences compared to the size of the text using AL-Definite words and the complete dataset.

Pre-processing: The newswire stories of the AFP were written in SGML format. The documents are pre-processed by (1) considering only three fields: document number, document title, and the body of the document. (2) Each document is divided into sentences, where the sentence is determined as the text between two successive ‘<P>’ tags. Still, if the sentence length is limited to 256 characters, it its length is longer, then it is divided into two or more sentences. (3) all of the remaining tags are removed, the punctuation and numerals, and the stop words are removed as well. (4) All the text is normalized by converting the forms of the letter ‘ALIF’ to ‘ALIF’ without ‘HAMZA’, and the ”TA’ Marboota” is converted to ”HA”’

After dividing the TREC dataset into sentences, it is found that the number of sentences formed using all words of the dataset is 3,118,047. When using AL-Definite words as a representative subset, it is 2,956,145 sentences. Although the number of sentences of the AL-Definite words is about 94.8% of the sentences using the whole dataset, all documents were represented by AL-Definite sentences. The size of the sentences is much smaller than the sentences of the complete dataset; the size of the text in the case of the AL-Definite sentences is about 30% of the size of sentences of the complete dataset; these statistics are presented in Fig. 3.

The following settings of the SkipGram model were used: word frequency selected to be ten as in^19^ and^22^, the size of the word vector is 300, and the window size is 5. The settings of the GloVe were: window size = 4, iterations = 10, and vector size = 5. Single embedding is applied to all experiments, and the most similar words to each word are determined using the cosine similarity function, and stored in an array. The trained word embedding models were used to test different scenarios of PRFQE, as follows: the effect of stemming of model training, the effect of the indexing method, the effect of the number of pseudo-relevant documents, the effect of the number of words selected from each document (extended words), the effect of the method in which top-m words from each document are selected; i.e., select top-m words from each document against selecting top-m words from the combination of all documents, the option of applying word embedding for only the original query is tested against applying embedding on the extended query, and finally, the effect of using larger dataset to train the word embedding models is tested by adding sentences from the Watan-2004^41^ dataset to the sentences obtained from the TREC dataset. These experiments are described as illustrated in Table 2.

The methods described in Table 2 are tested on the four options described in Table-1, and for the two word-embedding models (SkipGram and GloVe). The embedded words for each query word were stored in tables for each training method; a maximum of three closest embedded words were used because it has been found that it is a satisfactory size of a word context as in^11^.

The PRFQE methods were applied using a Java application that implements the indexing and search system, where each expansion method was iterated to expand two to five pseudo-relevant documents (the parameter d is variated from 2 to 5). The words were nested for each document iteration from 10 to 20 expansion words (the parameter m is variated from 10 to 20). All of the word embedding experiments were tested using the two closest embedded words (the parameter t=2) because it is empirically found that t=2 is the best value for this parameter. The experiments were applied to the 75 topics (or queries) of the TREC 2001/2002. Each iteration was evaluated for the Mean Average Precision (MAP), Precision at the tenth retrieved document (P10), and for the R-Precision, which is the precision at the R^th^ returned document where R is the number of the actual number of the relevant documents to each query, according to the relevance judgment of the TREC.

The indexing and search system was implemented such that stop words were removed, and words were normalized by applying the Light10 stemmer. The title and the description of each topic are used to form a query, and the retrieval is also enhanced by giving the title words of each topic double the weight of the description words.

**Table 2:** Pseudo-relevance query expansion schemes.

**Scheme**	**Description**
**SX**	Pseudo-relevance feedback without word embedding.
**ESX**	Embed words of the original query, and pseudo-relevance expansion
**SXE**	Pseudo-relevance expand query, embed the expanded query, and resubmit
**ESXD**	ESX where expansion is by adding top-m words from each pseudo-relevant document
**ESXA**	ESX where expansion is by adding top d´m words from a combination of all pseudo-relevant documents.

### Results and discussion

**Fig. 4:**
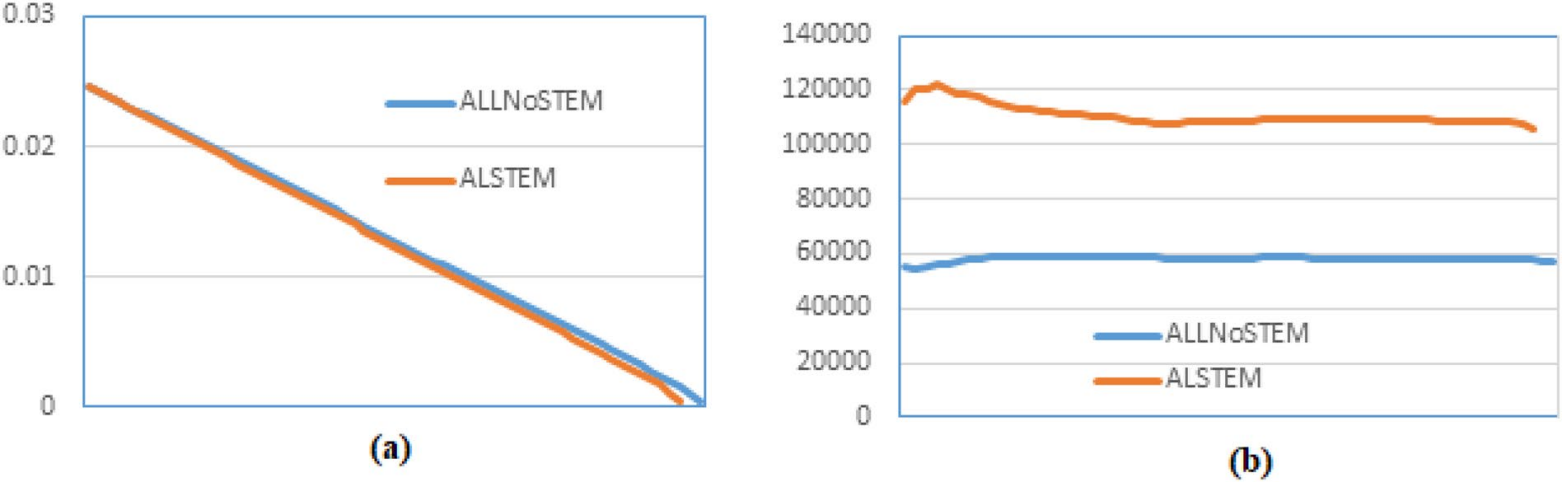
(**a**) Learning rate comparison (**b**) Words/second processing rate for ALSTEM vs ALLNOSTEM.

The main objective of this study is to shorten the training time of the word-embedding model by using less text while preserving the retrieval performance of the word-embedded PRFQE. To test the achievement of this objective, we presented a comparison between the learning rates of training the word embedding model using all words of the dataset and using just the AL-Definite words to train the model, and the result is illustrated in Fig. 4 (a). It can be noticed that there is a minor change in the learning rate between using a representative subset of words (AL-Definite words) and all of the words for training the model. Moreover, the rate of vocabulary construction (for the SkipGram as an example) trained using All-words (ALLNOSTEM) and using only AL-Definite words with stemming (ALSTEM) is illustrated in Fig. 4 (b), which shows a wide gap between the rate of word vectorizing in the two cases; i.e., the word vectorizing rate of using AL-Definite words as a representative subset is much faster than the word vectorizing rate of using the whole dataset words, as an example, training the model by ALSTEM gives an average of 83% enhancement over the rate in case of ALLNOSTEM. The training time is reduced to about 10% of the time needed to train the model using all words of the dataset and word vectorization rate because of the big difference in the text size of sentences of the two methods, as illustrated earlier in Fig. 3. These results show the effectiveness of using AL-definite words as a representative subset of words for training the word embedding models. However, this big difference between the learning and word vectorizing rates should not negatively affect the word embedding PRFQE’s retrieval results.

The following experiments were designed to examine the retrieval effectiveness for each training criterion using two different document indexing methods, namely: All-Words, in which all of its words index each document, and the AL-Before method, in which each document is indexed only by the AL-Definite words and words before them, as explained in^39^. The experiments are evaluated at different recall levels, and the results are presented using three diagrams: a diagram for the MAP, the P10, and the R-Precision.

The first experiment determines the effect of word embedding on retrieval by comparing PRFQE with and without word embedding and examines the importance of training the word embedding model using AL-Definite words as a representative subset instead of All-Words. In this experiment, two options for training the SkipGram embedding model are used; the first option is to train the model using All-words of the dataset, and the other option is to use only AL-Definite words as training text. Using the terminology of Table-1 and Table-2, this experiment compares SX and ESX (using ALLNOSTEM and ALNOSTEM).

**Fig. 5:**
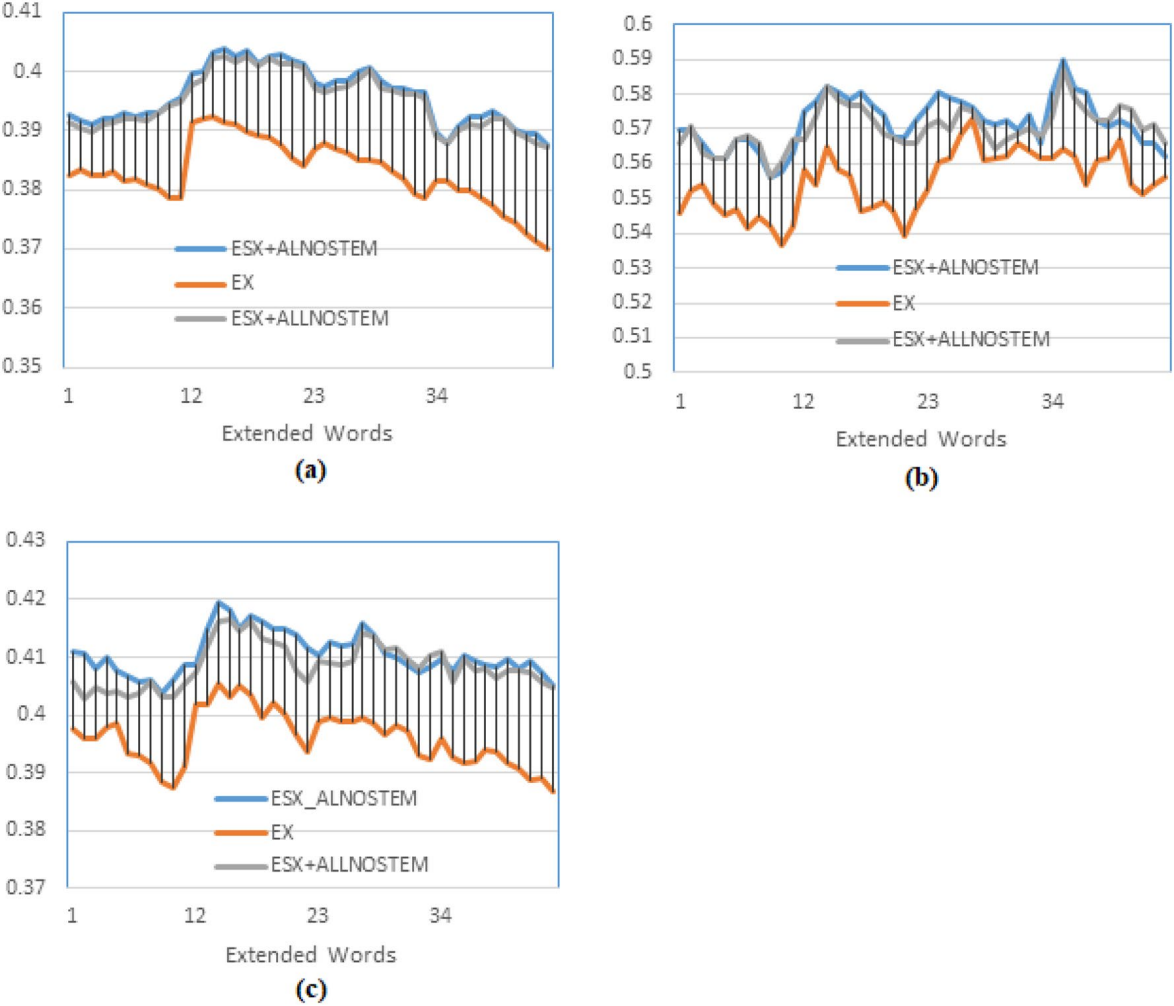
Testing the effect of using a subset of text to train the word embedding against using the whole dataset, and both against not using word embedding. The figure presents the precision comparison of PRFQE without word embedding against SkipGram word embedding query expansion. The model is trained by two data sets: AL-Definite words, and All-Words without stemming, and documents are indexed by the AL-Before method. (**a**) presents the Mean Average Precision, (**b**) presents the precision at the 10^th^ returned document, and (**c**) presents the precision at the R^th^ returned document.

The indexing method used is the AL-Before, and the results are presented in Fig. 5; each label on the x-axis of this figure represents an extended document, such that label ’1’ right-to label ’12’ means expand by two documents, label ’12’ right-to label ’23’ means expand by three documents, label ’23’ right-to label ’34’ means expand by four documents, label ’34’ right-to the end of the axis means expand by five documents, and for each expanded document, the extended words are varying from 10 to 20. This description of the x-axis is the same for all of the following figures.

It could be observed that the maximum MAP results for the three curves (in Fig. 5a) are obtained at the horizontal axis points that represent d=3 by extending the top three ranked documents, and at the same point that represents m=13; i.e., extending 13 words of each of the three documents. The two training options show similar maximum MAP (see Fig. 5a), and the maximum precision at P10 is the same as well, which is obtained at d=5 (as presented by Fig. 5b), but the R-Precision for the ESX+ALNOSTEM is better than that of ESX+ALLNOSTEM, and it is obtained at d=3, m=13, as in Fig. 5c. Moreover, using word embedding (ESX) shows an apparent enhancement (at the three recall levels) over PRFQE without word embedding (SX). The similar results obtained by training the word embedding model by all of the words (ALLNOSTEM) and training by only the AL-Definite words (ALNOSTEM) assure the representativeness of the AL-Definite words to the whole dataset. The two training methods show similar results at the three recall levels, that assure the stability of the results obtained by training the model by this subset of words while preserving the retrieval performance.

To give more evidence to accept these results, t-Test is used to examine the null-hypothesis that is ”there is no significant difference between expanding a query using word embedding, and the PRFBQE”. We calculated the paired t-test between the results of the 75 queries in the same conditions for both of these two methods, and it was 1.59102E-23, Which indicates rejecting the null hypothesis, meaning that word embedding provides a statistically significant improvement to the query expansion. On the other hand, when calculating the t-test between the results of using all of the terms, and the results of using the AL-definite words, the t-test value is 0.477074467, which indicates accepting the null hypothesis that is no significant statistical differences between using these two method for training the word embedding model. The latter result proves the validity of the assumption that using a representative subset of words is sufficient to train the model.

The explanation for obtaining the maximum P10 by extending the original query by five documents and 11 to 14 words for each document is that the probability of a relevant document (in the top-10) can be estimated by the P10 without query expansion, which is about 54% as presented in^39^, so it is most likely to bring more relevant documents in the top-5 returned documents. For example, the P@1 (the precision at the first retrieved document) without query expansion was 60.7%. However, it becomes 61.56% after a word-embedded query expansion, which means that word embedding assures that the first retrieved document is relevant.

**Fig. 6:**
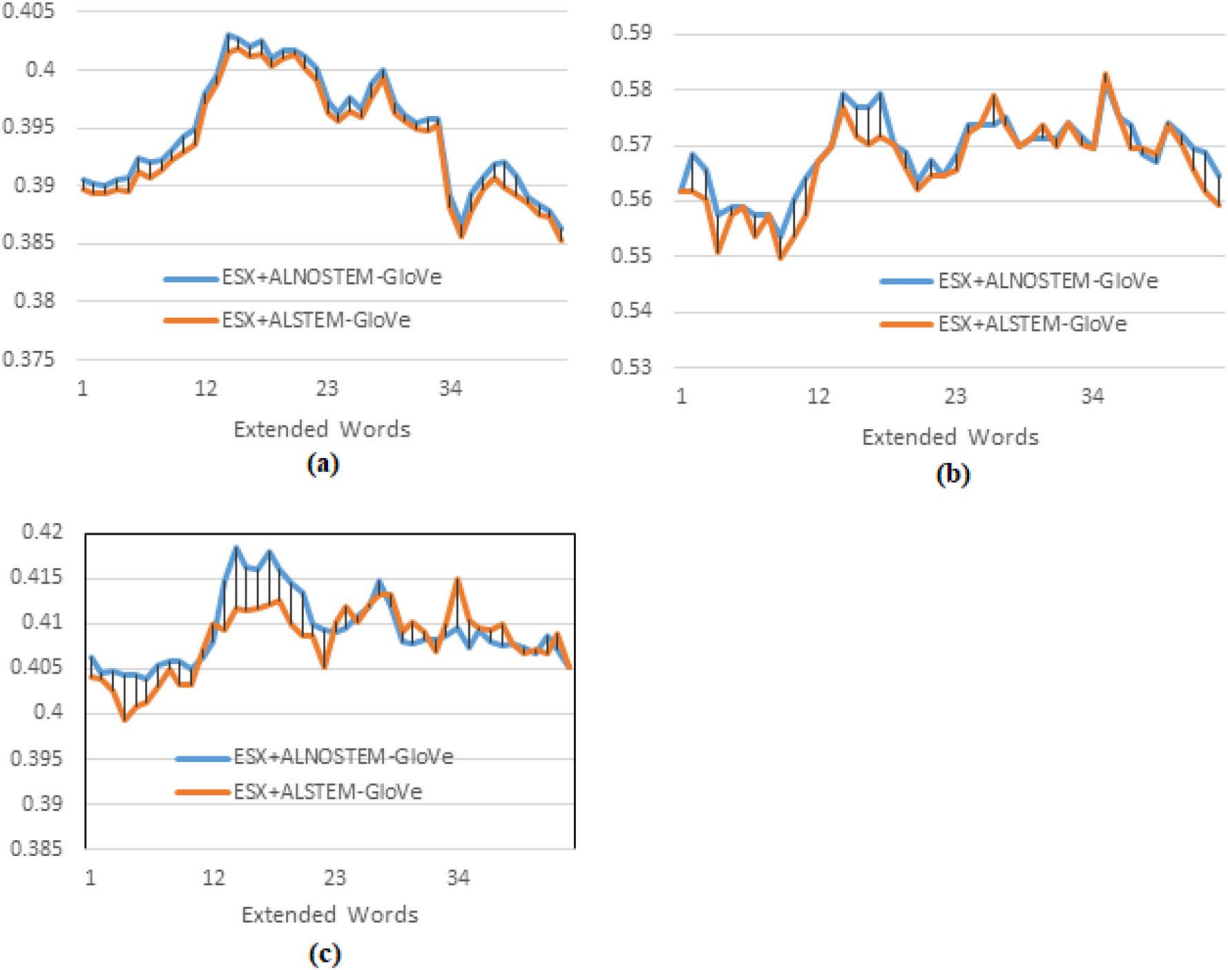
Testing the effect of training the word embedding model using stemmed text against not-stemmed text on the word embedding. The precision comparison of embedding similar words to only the original query, the GloVe word embedding model is trained using the AL-Words (stemmed and without stemming), and the documents are indexed using the AL-Before method.

The following experiment examines the effect of stemming on training the word embedding model, so the system is fed with embedded words resulting from training the GloVe model using the AL-Definite words, without stemming (ALNOSTEM), and AL-Definite words with Light10 stemming (ALSTEM), and the results were as illustrated in Fig. 6. The results show a slight enhancement of MAP for ALNOSTEM over stemmed text ALSTEM (Fig. 6 a); this slight difference is justified as ALNOSTEM shows better results for higher recall levels that are used to calculate MAP, which is consistent with the results concluded by other researchers, as in^5^ and^19^. On the other hand, ALSTEM shows better results in lower recall levels (at P10), where the maximum P10 is obtained at extending the original query by five documents, as shown in Fig. 6 b, which could be explained as in the previous experiment. It should be noted that the number of words for ALSTEM is about 60% that of ALLNOSTEM, which makes the training time shorter, so a trade-off between MAP and the training time could give more implementation options. The maximum R-precision for ALNOSTEM is higher than the maximum R-Precision for ALSTEM, as in Fig. 6 (c); it is obtained by extending the original query by the words of the top-3 returned documents, while the maximum R-precision of the ALSTEM is obtained by extending the query by the words of the top-5 documents.

**Fig. 7:**
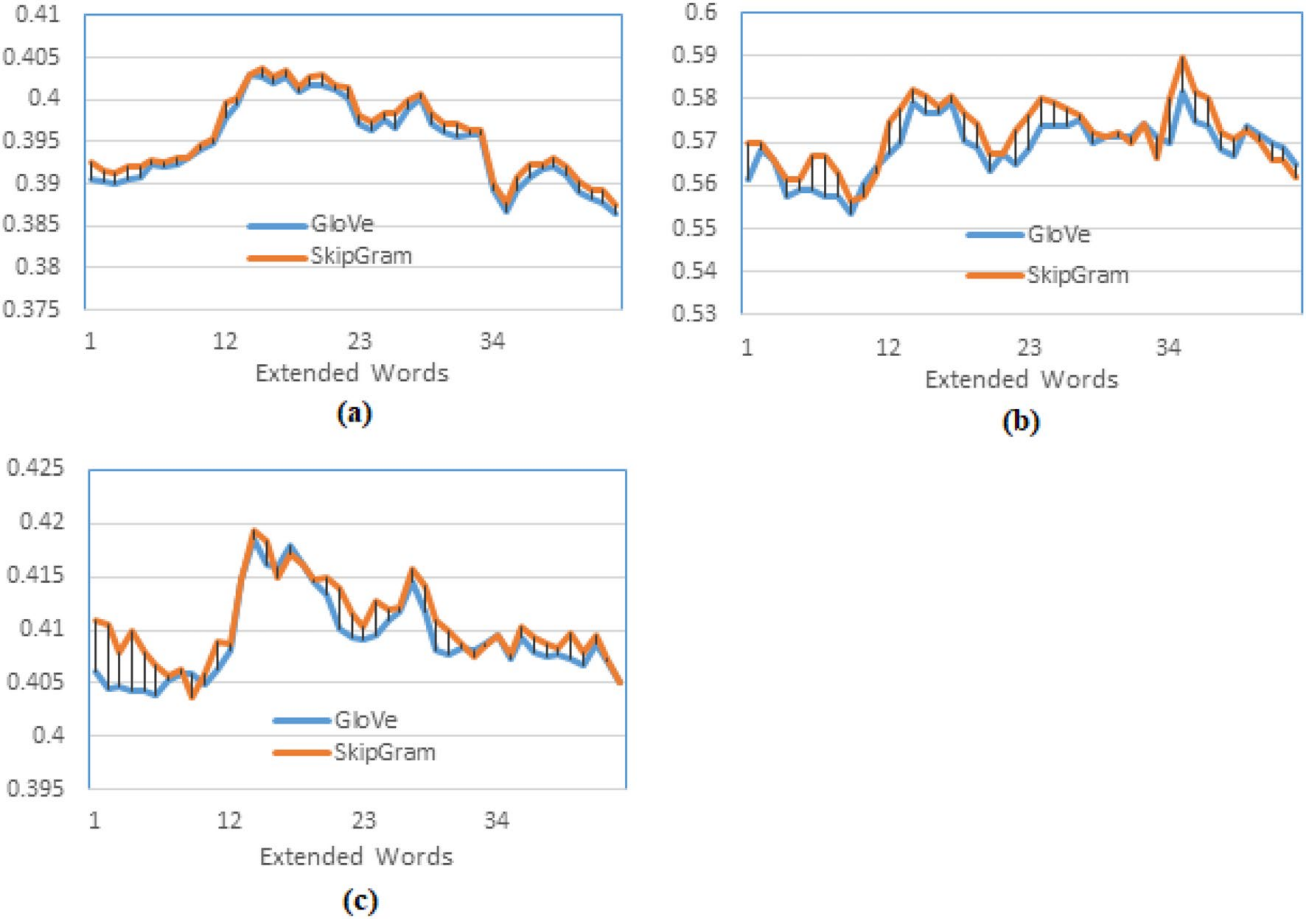
Testing the effect of the word embedding model on the pseud-relevance feedback after word embedding, comparing query expansion using word embedding of SkipGram and GloVe models, both are trained on ALNOSTEM and documents are indexed using the AL-Before indexing method.

Using different word embedding models and PRFQE shows insignificant differences regarding the MAP results; for example, the results of SkipGram and GloVe for the same query expansion scheme are represented in Fig. 7 (a). This result is relevant to the results reported by other researchers, such as^3^. However, there are some differences in P10, as in Fig. 7 (b). The slight difference of P10 showed by the SkipGram over the GloVe can be explained by the fact that GloVe determines the similarity between words on a global co-occurrence within a dataset. In contrast, the SkipGram determines this similarity based on local co-occurrence within a text. Hence, the results become closer at higher recall levels, such as R-Precision, as presented in Fig. 7(c).

**Fig. 8:**
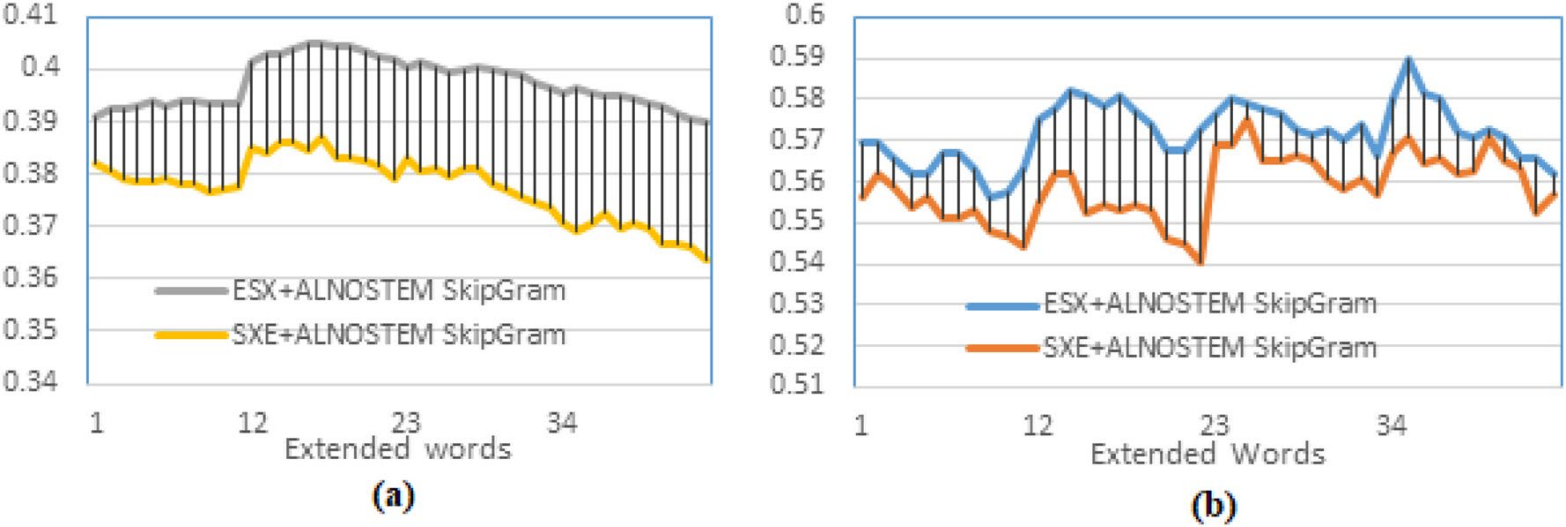
Testing the effect of word embedding expansion before pseudo relevance feedback (the original query) against word embedding expansion of the expanded query, (**a**) MAP comparison between applying word embedding for the original query words (ESX), and applying word embedding for the expanded query (SXE), (**b**) Precision at 10th returned documents (P10).

The following experiments examine different options for applying word embedding to the PRFQE. The first experiment in this context compares applying the word embedding to the original query, i.e., before PRFQE (ESX), which is the first scheme described in Sect. "Pseudo-relevance feedback", and the application of word embedding to the expanded query (SXE), the second scheme described –also– in section 4.2. The results presented in Fig. 8 (a) show that embedding the original query (the first scheme) has better MAP than embedding the expanded query (the second scheme), and the P10 of ESX is better than its value for the SXE as presented in Fig. 8 (b). This result satisfies the last assumption (point 4) listed at the end of Sect. "Pseudo-relevance feedback". It can be explained as the expanded query (in SXE) having words added from the pseudo-relevant documents, which itself could have a different meaning than the original query words, and the similar embedded words of these expanded words most likely to have a different meaning than the words of the original query. A closer look at Fig. 8 (a) shows that as the number of extended words increases, the difference in the MAP becomes more pronounced, indicating that as more words are added, they carry a different meaning than the words of the original query. Another experiment examines the effect of selecting top-m weighted words from each distinct pseudo-relevant expanded document (ESXD) against selecting the top m$\times$d weighted words after mixing m words from each of the d pseudo-relevant expanded documents (ESXA), as explained in Sect. "Pseudo-relevance feedback".

**Fig. 9:**
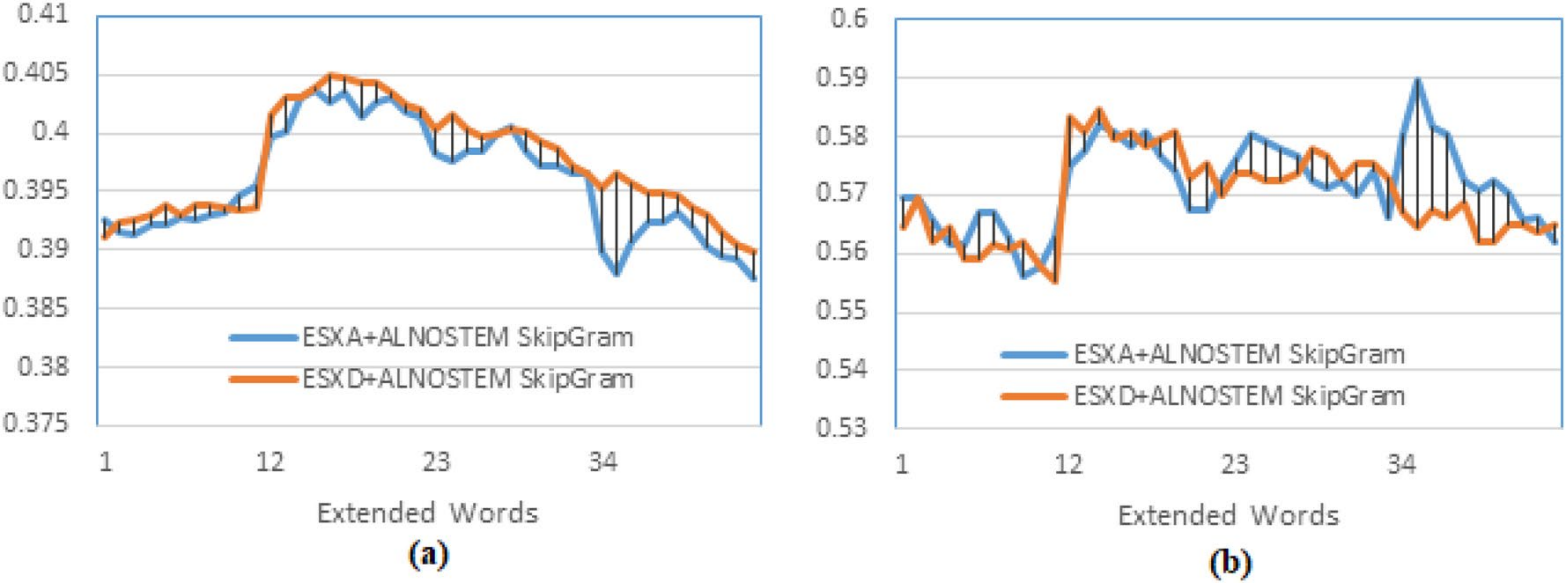
Testing the effect of the method of selecting the expansion words by comparing the MAP of expanding the original query by the top-m words from each of the top-d returned documents (ESXD) against expanding each query by m*d top words after mixing words of all top returned documents (ESXA).

**Fig. 10:**
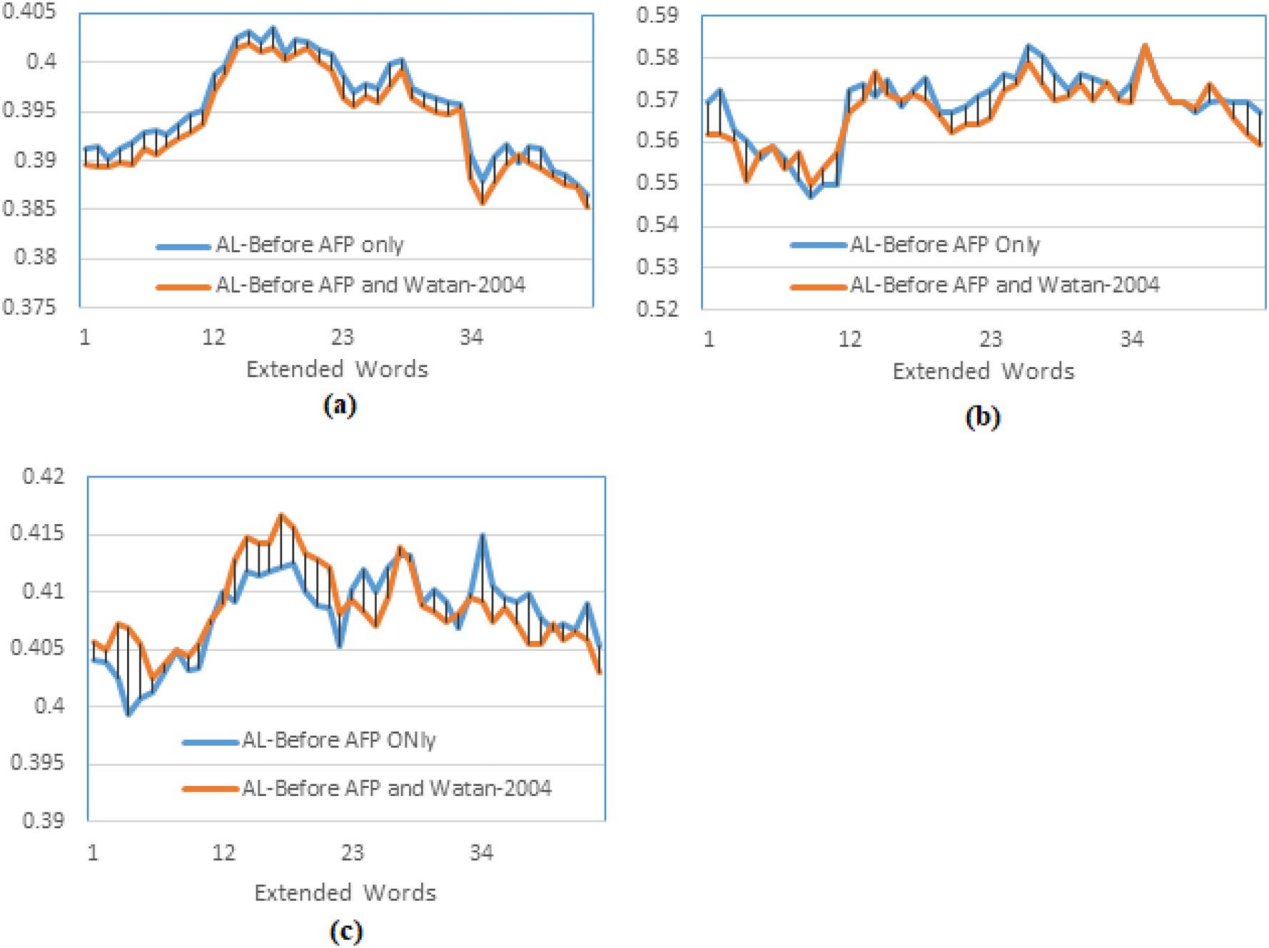
Teasing the effect of using more text to train the word embedding mode. This figure presents a comparison of the precision of training the GloVe model using a combination of two datasets TREC 2001/2002 (AFP news documents) and Watan-2004 dataset, against using a single dataset (AFP), the text in both cases is stemmed using the Light10 stemmer. (**a**) MAP, (**b**) Precision at the 10^th^ returned document, (**c**) Precision at R-Precision.

The result of this experiment is plotted in Fig. 9. It could be observed that the maximum MAP for the ESXD is slightly higher than the MAP of the ESXA for most of the values of d and t (Fig. 9 (a)), and the maximum MAP for both of the methods found at d=3, but the maximum P10 of the ESXA is higher than that of the ESXD (see Fig. 9 (b)). It is found at d=3 for the ESXD and at d =5 for the ESXA. The result can be explained as the ESXA adds top weighted words (globally) from a combination of the pseudo-relevant documents; it is more probable to have the right similar semantic words within this combination since the top-weighted words are most likely to be repeated in more than one document, making them topic-specific words. On the other hand, in the ESXD, the top-weighted words are determined (locally) in each separate document that could be irrelevant to the query. However, for higher recall levels, it is more probable to find returned documents having the exact words but of different semantics, which explains the lower values of the MAP of the ESXA. For more testing of the proposed model training method, more experiments were applied to test the effect of using more than one dataset and two different indexing methods. The word embedding models trained by the text of both the TREC 2001/2002 (AFP news) and the Watan-2004 datasets. The watan-2004 (https://sites.google.com/site/mouradabbas9/corpora/text-corpora?authuser=0) dataset has about 20291 news documents of the Al-Watan Omani newspaper, distributed over six topics, prepared by Murad Abbas^41^. It is about 114 MB in size. The word embedding models (GloVe and SkipGram) are trained using sentences that contain only the AL-Definite words from a combination of the stemmed text of these two datasets (ALSTEM), where the documents are indexed using the AL-Before indexing method. The results of the word-embedded PRFQE are presented in Fig. 10.

It could be observed from Fig. 10 (a) that the MAP of training the Glove model with the AFP only has slightly higher values than training the model using both of the datasets. However, at lower recall levels, the maximum precision at P10 and R-Precision of applying both datasets show better results, as presented in Fig. 10 (b) and (c), respectively.

**Fig. 11:**
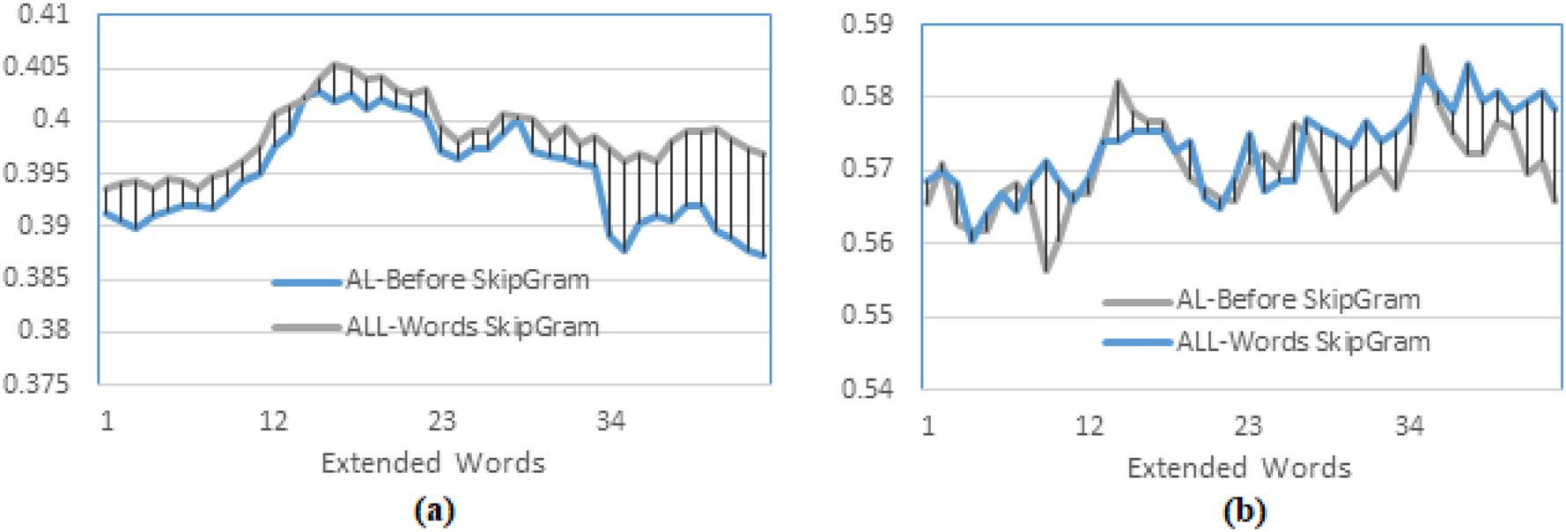
MAP of the PRFQE in which the SkipGram word embedding model is trained using both of the AFP and the Watan-2004 datasets, the sentences used for training the model are formed using the ALNOSTEM, the documents are indexed using two methods: AL-Before and All-Words methods, (**a**) comparing the MAP of the two indexing methods, and (**b**) the P10 of the two indexing methods.

Initial results indicate that training the model on a larger volume of text of the same type (news documents from the same period) improves precision at lower recall levels, but has a negligible effect on the overall recall space. This outcome is justified by the datasets’ differing focuses: the Watan-2004 documents concentrated on local news from Oman and the Gulf countries, while AFP maintained an international focus. Consequently, the added words with similar global semantics increased precision at lower recall levels, but the words related to local Gulf semantics negatively impacted the overall recall performance (as presented in Fig. 10 (a)). A subsequent experiment applied the SkipGram word embedding model, trained using both the AFP and Watan-2004 datasets, and tested the word-embedded PRFQE (Pseudo-Relevance Feedback Query Expansion) on two indexing methods: AL-Before and All-Words. The Mean Average Precision (MAP) and Precision at 10 (P10) results are plotted in Fig. 11. The All-Words indexing method showed a slight improvement in maximum MAP over the AL-Before method, with the maximum MAP for both achieved at $d=3$. Conversely, the AL-Before method yielded a higher maximum P10, which was obtained at $d=5$, as seen in Fig. 11 (b). The All-Words method exhibits better MAP because its index includes all words, increasing the probability of finding semantically related words (to the query words) that match or co-occur with documents at higher recall levels; this justifies the performance gap as more extended documents are used (Fig. 11 (a)). In contrast, the AL-Before method’s superior maximum P10 is due to the SkipGram model being trained exclusively on AL-Words, making it more probable for the expanded AL-Words to be similar to the AL-Words embedded in the original query. The successful concept of using a representative word subset motivates further research to define additional conditions for finding optimal subsets in different datasets. Crucially, leveraging a subset of the data to train a word-embedding model accelerates the development and implementation of Arabic natural language applications, consequently shortening the time required to improve search engine results through query expansion.

On the other hand, it could be noted that using a subset of a dataset to train the word embedding model, to expand queries has some limitations; such as: First: the result of the P10, for example, is the same as the result of using the whole dataset just at some experimental setting of the PRFQE, it needs more experimental investigation to find that situation, in which the selected subset outperforming the whole dataset, as could be seen in Fig. 11 (b). Second: the best result of the MAP of all scenarios of PRFQE was achieved at different experimental settings, the number of pseudo-relevant documents, and the number of top-rated terms selected from each of these documents. Consequently, the right situation depends on the type of the desired dataset, the scheme described in this paper, and successfully tested for news-wire datasets, which contain documents of different topics, other datasets having documents of a limited number of topics could need some other treatment. Regarding the issues of morphological errors, out-of-vocabulary, and corpus bias, the morphological errors, for example, have a little impact on the retrieval results, because the newswires of the TREC corpus were written by expert journalists, and reviewed by editors, so there is a low chance to have this type of error, and the misspelled words are rare and it will have low weight, consequently it will not have considered to expand a query. Out-of-vocabulary has a minimal effect because the criteria of selecting the representative subset of words, indicate that the union of contexts of all of the selected words is the dataset itself, as explained in Sect. "Training the word embedding model". Bias corpus could occur because the TREC was written in standard Arabic, which is the form that all Arab world can understand, so the text written in other local forms could have different results.

### Comparison to other works

To compare the results obtained by the proposed methods in this paper to those of other related works, we summarize the maximum results gained for three different recall levels as in Table 3. The percentage of the enhancement is used for comparison since each work could have a different implementation environment.

**Table 3:** A statistical summary of the results obtained by the implemented methods, compared to the baseline.

**Method**	**Results**			% enhancement to baseline			% enhancement to PRFQE		
	MAP	P10	R-P*	%MAP	%P10	%R-P	%MAP	%P10	%R-P
AL-Before without query expansion	36.3	53.3	37.6	9.01	9.67	6.52	−3.94	3.50	−4.33
AL-Before PRFDQE without word embedding	39.2	56	40.6	17.72	15.23	15.01	3.73	8.74	3.31
AL-Before PRFQE with word embedding	40.37	**58.96**	**41.94**	21.23	**21.32**	**18.81**	6.83	**14.49**	**6.72**
All-Words without query expansion*	33.3	48.6	35.3	0.00	0.00	0.00	−11.88	−5.63	−10.18
All-Words PRFQE without word embedding*	37.79	51.5	39.3	13.48	5.97	11.33	0.00	0.00	0.00
All-Words PRFQE with word embedding	**40.53**	58.59	41.85	**21.71**	20.56	18.56	**7.25**	13.77	6.49

* R-P: R-Precision.

** Baseline All-Words indexing with Light10 stemming, no extra weight to title words, DF=1 included. Other methods exclude DF=1. Bold indicates best results.

**Fig. 12:**
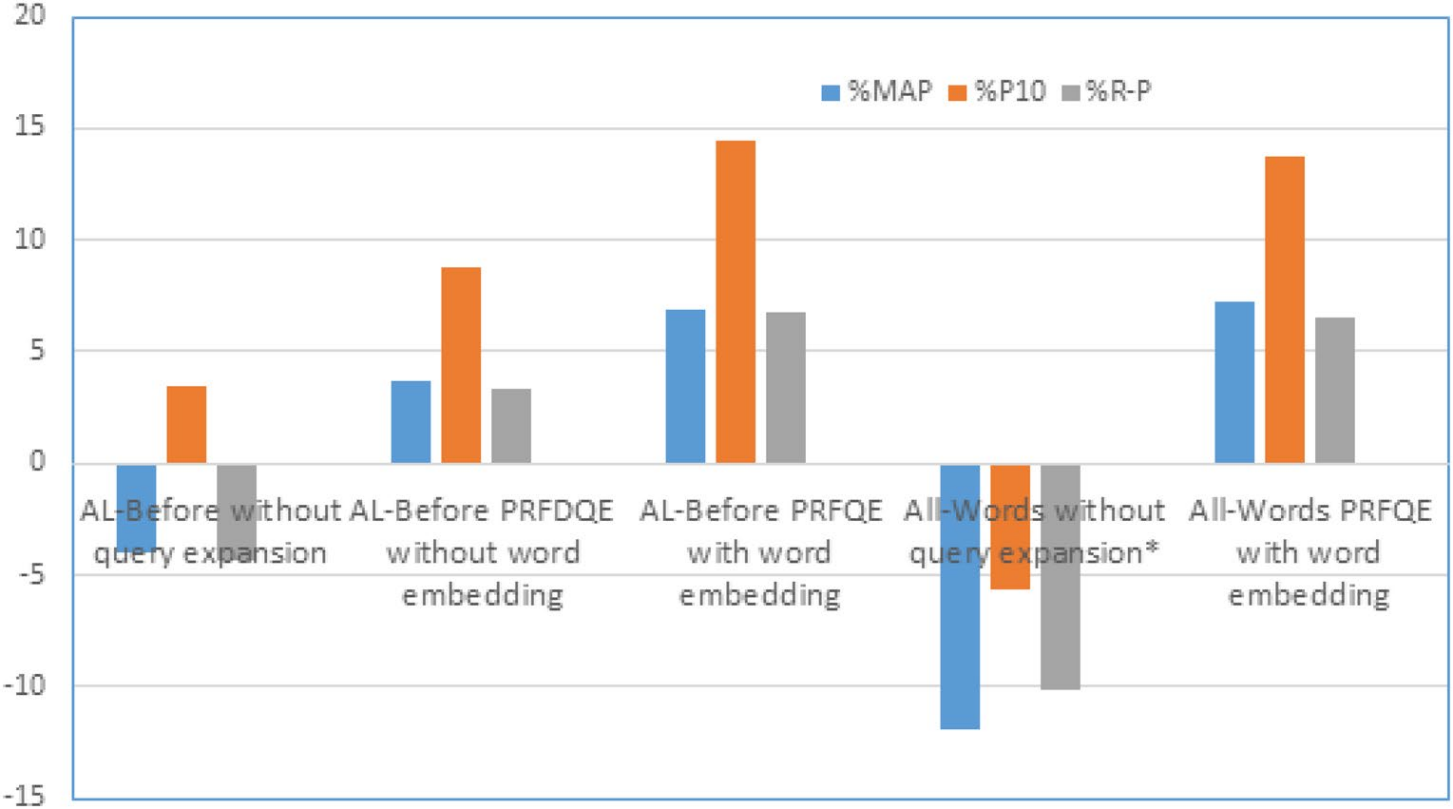
percentage of enhancement over the baseline: PRFQE without word embedding.

From Table 3, the MAP of using word embedding with the All-Words indexing method has a 7.2% enhancement over PRFQE without word embedding, and it has a 21.6% MAP enhancement over the basic All-Words without query expansion. The average precision at the 10^th^ returned document (P10 for the All-Words indexing method) is enhanced by 20.5% over the baseline indexing and 13.7% P10 enhancement of the word-embedded query expansion over the PRFQE without word embedding. The percentage of enhancement over the baseline is plotted in Fig. 12.

The most related work to the method proposed in this paper is El Mahdaouy et al.^19^ since they used the same dataset and evaluation metric. In that work, the maximum MAP is 41.11, and the maximum P10 is 55.07, while the MAP is 3% greater than the maximum MAP gained by the methods proposed in this paper, but the P10 of this paper is 7% greater than the P10 of the method proposed in^19^. Moreover, the word discovery rate of this paper is 83% greater than the word discovery rate of^19^, see Fig. 4(b), and the training time is shortened to 10% of the SkipGram, for example.

Farhan YH et al. in^24^ used the same dataset for evaluation; their method showed about 43.5% P10 for the TREC 2001/2002, less than the P10 shown by the proposed method in this paper. Hiba ALMarwi et al. in^1^ evaluated their proposal at the query level. They gave precision to each query and did not include an average or MAP value for all the queries. Recently, Farhan YH et al. in^15^, achieved a MAP of 39.8 by combining the Embedding-based Query Expansion (EQE1) and the DMNs, and the best P@10 was 52.4.

Ahmed Cherif Mazari and Abdelhamid Djeffal in^13^ used a semantic tree to reform the pseudo-relevance expanded query. They evaluated their proposed method using the Arabic BBC News corpora, and their results show a MAP of 0.524 at P10, which is a 5.8% improvement over the baseline of their test.

## Conclusion

This paper proposes an efficient Arabic query expansion scheme based on word embedding, such that the word embedding models are trained by using a representative subset of words instead of using the whole dataset, decreasing the training time and, at the same time, preserving the retrieval efficiency. Different alternatives of Arabic query expansion were examined: embed the original query, embed the expanded query, expand selected words from each distinct document, or select words from a combination of all of the returned documents to gather, and for two different indexing schemes (AL-Before and All-Words).

The results show that the subset of AL-Definite words is representative of an Arabic dataset to train a word embedding model, as it satisfies the conditions given in definition-1 and shows efficient retrieval. There is no significant difference between the retrieval of the word embedding trained by AL-Definite words and the word embedding query expansion trained by using the whole dataset words. Moreover, training a model using AL-Definite words shows better precision at lower recall levels, for example, at P10. Training the model using a stemmed text shortens the training time without affecting overall retrieval, but could improve retrieval at lower recall levels. The word embedding model does not significantly affect overall average precision, but could make a difference at lower recall levels.

The results show that embedding the original query words is better than embedding the words of the expanded query, i.e., word embedding should precede PRFQE. There is no significant difference (regarding the MAP) between expanding the embedded query by words from a combination of the top-weighted returned documents and selecting top-weighted words from each document. However, it is better to select the expanded words from a combination of top-similar returned documents at lower recall levels.

In general, systems concerned with better retrieval at lower recall levels should expand the query by combining or re-weighting words from more top-returned documents. The P10 could estimate this number the system shows before PRFQE. Using different data sets to train the word embedding model could improve retrieval at lower recall levels, but could negatively affect overall average results. In future work, we will apply the proposed method of representing a dataset by a subset of words for training a transformer-based word embedding, such as BERT, to reduce the training time and to develop some schemes to use these models for more efficient keyword-based PRFQE. Moreover, the idea of the embedding theorem of Takens^42^, which states that a one-dimensional chaotic time series could be considered a compression of a higher-dimensional chaotic series. According to this idea, a representative subset could be viewed as a compression of the whole dataset. This idea could be applied if we confirm that a representative subset is chaotic.

## Data Availability

All datasets used are publicaly available and the sources are properly cited in the paper.
